# Sex Differences in Copper Concentrations during a Sports Season in Soccer Players

**DOI:** 10.3390/nu15030495

**Published:** 2023-01-18

**Authors:** Víctor Toro-Román, Diego Muñoz, Marcos Maynar-Mariño, Sara Clemente-Gil, María C. Robles-Gil

**Affiliations:** School of Sport Sciences, University of Extremadura, 10003 Cáceres, Spain

**Keywords:** plasma, urine, erythrocytes, platelets, soccer

## Abstract

Physical training produces changes in the concentrations of trace mineral elements. Sex differences in copper (Cu) concentrations in athletes are scarce. The objectives of this study were (i) to analyze changes in intracellular (erythrocytes and platelets) and extracellular (plasma and urine) Cu concentrations during a sports season in soccer players and (ii) to analyze sex differences. A total of 46 soccer players (22 men and 24 women) participated in the study. Three assessments were performed throughout the sports season. Anthropometry, body composition, nutritional intake, physical condition, female hormones (menstrual cycle) and hematology were evaluated, as well as Cu determination (plasma, urine, erythrocytes, and platelets). Regarding longitudinal differences, there were discrepancies in plasma, urine, absolute erythrocyte, and absolute platelet Cu concentrations (*p* < 0.05). There were differences between sexes in Cu concentrations in urine, erythrocytes relative to cell number and in platelets relative to cell number (*p* < 0.05). During a sports season, there are changes in Cu concentrations in soccer players. Likewise, there could be sex differences in urinary, erythrocyte and platelet Cu concentrations.

## 1. Introduction

Copper (Cu) is an essential trace mineral element (TME) in humans present in +1 and +2 states. Therefore, its main function involves oxidation–reduction reactions [1]. Cu plays an important role as an enzymatic cofactor in cuproenzymes, Cu-dependent oxidases involved in various reactions [2]. The human body contains about 1.6 mg Cu/kg body mass with different distributions among tissues, organs, and blood. Bone contains 40% of body Cu, while muscle tissue contains 23%. With respect to organs, the concentrations in the kidney, 12 mg/kg; liver, 6 mg/kg and brain, 5 mg/kg, stand out. Blood contains approximately 6% of total body Cu [3].

Cu is involved in various aspects of energy metabolism. It is an important component for the synthesis of hemoglobin, myoglobin, cytochromes and peptide hormones [4]. In addition, it is necessary for the optimal utilization of iron (Fe) [5] and protects against the formation of reactive oxygen species (ROS) through the action of superoxide dismutase (SOD) [6]. In relation to the above, ceruloplasmin, a Cu transporter, participates in the binding of free Cu ions preventing oxidative damage [7].

In situations of high metabolic demand, circulating and cellular TME deficits may affect athletic performance [8]. Metabolic adaptations to physical training could be modulated by the nutritional status of TME [9]. It is known that the level of training and the practice of physical exercise produce changes in Cu concentrations. For example, serum Cu levels increased by 37.3% in trained and 21.5% in untrained men after a maximal incremental test [10]. However, urinary Cu excretion decreases on rest days compared to physical training days [11]. Decreases in urinary Cu concentrations have been observed after a period of training [12].

Moreover, the effects of TME on the organism could manifest differently between sexes. This could be due to differences in the kinetics and mode of action of TME [13]. Among the factors that may have an influence are changes related to menarche, pregnancy, lactation and menopause [14]. The sex influence on extracellular Cu concentrations has been previously analyzed. It is known that women show higher serum and urinary Cu concentrations compared to men [15,16,17]. However, research focuses on the general population and not on athletes.

A large number of studies use cuproenzymes to assess Cu status [18]. However, it is accepted that response capacity and ceruloplasmin concentration can be affected by a variety of factors, such as age and sex [7]. Milne et al. [19], in 1998, concluded that a lower concentration or a change in a single index may be inadequate to assess nutritional status. Therefore, it is recommended to use several parameters simultaneously to analyze Cu status [20].

Research on the influence of physical training focuses on individual or cyclic sports [12,21,22,23]. In soccer players, research is more scarce. In relation to the above, previous authors reported increases with respect to basal values in Cu concentrations after the end of a soccer match regardless of the time of the match (morning or evening) [24]. However, Metin et al. [25] reported that soccer players presented lower plasma Cu concentrations in relation to the control group, being in the same line as that reported by Toro-Román et al. [20]. However, as mentioned in the previous paragraph, sex differences in TME concentrations have not been studied in athletes. Therefore, the aims of the present study were (i) to analyze the changes in intracellular (erythrocytes and platelets) and extracellular (plasma and urine) Cu concentrations during a sports season in soccer players and (ii) to analyze the differences between the sexes in soccer players.

## 2. Materials and Methods

### 2.1. Study Design

The present longitudinal quasi-experimental study lasted approximately 11 months. Three assessments were carried out during a regular soccer season. The first assessment (1st assessment) was carried out the week of the beginning of training (August). The second assessment (2nd assessment) was carried out in the middle of the season, after the end of the first round of the regular season (January). The third assessment (3rd assessment) was carried out during the last week of training for both teams (May–June).

All assessments were carried out the same week of each month, in the morning and in the same order for all participants to avoid the effects of circadian cycles. Comparisons were made between assessments (longitudinal; time effect) and between groups (cross-sectional; sex effect).

### 2.2. Participants

The sample participating in the present investigation consisted of a total of 46 soccer players. All participants were informed about the purpose of the study and signed a consent form before enrollment. The protocol was reviewed and approved by the Biomedical Ethics Committee of the University of Extremadura (Cáceres, Spain) (code 135/2020) following the guidelines of the 1964 Helsinki ethical declaration.

Subjects were divided by sex: Men soccer players (n = 22) and women soccer players (n = 24). Men soccer players belonged to a semi-professional soccer team from the fifth category of Spanish soccer. The women soccer players belonged to a second category Spanish soccer team which competed in the 2nd division at the national level. All participants trained and played league matches in the same city. The characteristics of the teams are shown in Table 1.

According to the Spanish sports statistics yearbook 2022, the number of Extremadura soccer federation licenses is 18,746. Therefore, the sample of the present study would be adequate considering a confidence level of 85% and a margin of error of 10%.

The inclusion criteria for participation in the present study were: (i) Residence in the same city 1 month before and during the study; (ii) not suffering from any type of illness; (iii) not taking medication or supplementation that included TME during the study period or the month prior to the first evaluation; (iv) not smoking or using drugs; (v) more than 5 years’ experience competing in soccer; (vi) not modifying nutritional and physical activity habits during the study; and (vii) not going more than 30 days without training with the team. In addition, the women had to meet the following inclusion criteria: (viii) Have regular menstrual cycles for at least six months prior to the start of the study and during the study; (ix) not suffer from problems related to the menstrual cycle; and (x) not use contraceptive methods.

The coaching staff of each team provided information on player characteristics, training, matches and injuries with the consent of the participants. The internal load data have not been added since each coaching staff used a different methodology. Physical activity was assessed using the International Physical Activity Questionnaire—Short Form (IPAQ-SF) Spanish version [26].

### 2.3. Menstrual Cycle

To learn about the characteristics of the menstrual cycle, women soccer players completed an online questionnaire. A researcher was available to help participants respond. The questionnaire consisted of questions about cycle length, duration of bleeding, type of bleeding, age of onset, regularity of menstruation, and related pain or symptoms. It is important to know the characteristics of the menstrual cycle as some studies have noted that there may be variations in the concentrations of certain TME throughout the cycle [27,28]. Therefore, all assessments, as far as possible, were performed at the same phase of the menstrual cycle. In addition, as developed below (Table 2), the female hormones progesterone and estradiol-17β concentrations were determined.

### 2.4. Anthropometric and Body Composition

Anthropometric evaluations were carried out with subjects fasting and wearing as little clothing as possible according the guidelines of Porta et al. [29]. The following assessments were performed: stretch stature, body mass, skinfolds (abdominal, suprascapular, subscapular, subscapular, tricipital, thigh and calf), breadth (biscipital, humerus and femur) and girth (relaxed arm and calf). All assessments were performed on the right side of the participants.

The materials used were a wall-mounted stadiometer (Seca 220. Hamburg, Germany), an electronic digital scale (Seca 769. Hamburg, Germany), a Holtain© 610ND skinfold compass (Holtain, Crymych, UK), a Holtain© 604 pachymeter (Holtain, Crymych, UK) and a Seca© 201 tape measure (Seca, Hamburg, Germany). Three evaluations were performed for each parameter and the mean was chosen for statistical analysis. The parameters of fat, muscle and bone percentage were obtained using the formulas of the Spanish Group of Kinanthropometry [30]. The Yuhasz equation was used to calculate the fat percentage [31]. The muscle percentage was obtained by dividing the muscle weight, obtained by subtracting the body, bone (Von Doblen equation modified by Rocha [29]), fat and residual (Wurch equation [29]) weights, with the total weight and divided by 100.

### 2.5. Physical Fitness

All tests were performed in the morning, after blood draws and after a free breakfast.

Lower body muscle power was evaluated by means of vertical jump tests. Squat jump (SJ) and countermovement jump (CMJ) tests were performed (Optojump, Mycrogate, Mahopac, New York, NY, USA) [32]. The warm-up consisted of performing knee and hip mobility. After that, the participants performed half squats without additional body mass and then an isometric squat for 5 s. Two attempts at each jump were performed and the best one was chosen for statistical analysis. There was 30 s of recovery between jumps.

For the SJ, participants initiated the movement from a squat position with the knees at a 90° angle with the arms resting on the hips. After 3 s of holding the squat position, subjects performed a jump without countermovement at maximum intensity.

For the CMJ, participants started the execution from an upright position, with feet shoulder-width apart and hands resting on the hips. Subjects performed a knee flexion-extension followed by a jump of maximum possible intensity.

To assess maximal aerobic capacity, a maximal incremental test was performed. The test was performed on a treadmill (Ergofit Trac Alpin 4000, Pirmasens, Germany), equipped with a gas analyzer (Geratherm Respiratory GMBH, Ergostik, Ref 40.400, Corp, Bad Kissingen, Germany) and a Polar heart rate monitor (Polar^®^ H10, Kempele, Finland). The protocol consisted in running in 1-min stages until voluntary exhaustion of the subject. The test started at 7 km/h and increased by 1 km/h every minute at a steady 1% gradient. Prior to the test, a 15-min warm-up was performed at 6 km/h on a treadmill.

### 2.6. Nutritional Intake

This section is similar to that reported by Toro-Román et al. [33]. To know the intake of macronutrients and Cu, the participants filled out a nutritional questionnaire. The nutritional composition of each food was assessed [34]. Participants were given a document where they had to indicate the amount and frequency of food intake for the 3 days prior to the assessments. The investigators performed a food conversion to estimate consumption using predetermined tables [34].

### 2.7. Sample Collection

On the days prior to the evaluations, the physical trainers of each team were provided with the urine kit (container + tube) to distribute to the players. Extractions began around 8:00–8:30 a.m. Subjects came to blood collection with samples of the first urine of the day. Urine was collected by participants in 100 mL containers. Urine samples were transferred to 9 mL BD Vacutainer^®^ (Franklin Lakes, NJ, USA) tubes and frozen at −80 °C until analysis.

Fasting blood samples were collected. A total of 12 mL of blood was drawn using a 20 mL plastic syringe (Injekt, Braun, Melgunsen, Germany) and a sterile needle (Mirage Pic Solution, Trieste, Italy). Of the total, 2 mL were collected in Vacutainer^®^ tubes with a clot activator applied to determine hematological parameters (Coulter Electronics LTD, Model CPA; Northwell Drive, Luton, UK) and female hormones by the ELISA (enzyme-linked immunosorbent assay) technique with a spectrophotometer.

Furthermore, 8 mL were used to determine Cu concentrations in the different biological matrices. Two 4 mL BD Vacutainer^®^ tubes with sodium citrate were collected. For plasma, a Vacutainer^®^ tube with sodium citrate was taken and centrifuged at 1800 rpm for 8 min. The platelet-rich plasma was collected in dry BD Vacutainer^®^ tubes without additives and centrifuged for 10 min at 3000 rpm. The plasma was aliquoted into 1.5 mL Eppendorf tubes and allowed to stand at −80 °C. For mixing, 1 mL of pure water (Mili-Q) was added to the adherent platelets and vortexed (Cole-Parmer™, Stuart™, Vernon Hills, IL, USA). The mixture was transferred to an Eppendorf tube and stored at −80 °C. Erythrocytes were removed from the remaining blood and washed three times with 0.9% sodium chloride (NaCl). They were then collected in 1.5 mL Eppendorf tubes and stored at −80 °C.

### 2.8. Cu Determination

The techniques to obtain Cu in the different compartments are similar to those reported in other studies [20,33].

The determination of trace mineral elements was performed by inductively coupled plasma mass spectrometry (ICP-MS) (7900; Agilent Tech., Santa Clara, CA, USA). The linearity of the calibration curves for indium (In) were greater than 0.985. The values of the standard materials for this element (10 μg/L) coincided with intra- and interassay coefficients of variation of less than 5%.

For plasma and urinary samples, the reagents used were 69% nitric acid (TraceSELECT™, Fluka™, Madrid, Spain) and ultrapure water obtained from a Milli-Q system (Millipore^®^, Burlington, MA, USA). A rhodium dilution of 400 µgL-1 was used as the internal standard and continuously fed into the apparatus with the aid of the three-channel peristaltic pump. From the 0.20 mL of samples, a volume of 5 mL was made up with a 1% nitric acid solution prepared from a commercial one of 69% (TraceSELECT™, Fluka™, Madrid, Spain). The equipment was calibrated with several standards prepared from commercial multi-elemental dilutions of certified standards.

For erythrocyte and platelet samples, the reagents used in method development and sample analysis were nitric acid 69%, hydrogen peroxide (TraceSELECT™, Fluka™, Madrid, Spain) and ultrapure water obtained from a Milli-Q system manufactured by Millipore (USA). A 400 µgL-1 solution of yttrium and rhodium was used as the internal standard.

Samples were weighed on a precision balance and transferred to glass tubes for microwave digestion, and 3.5 mL of a 3:1 mixture of 69% nitric acid (TraceSELECT™, Fluka™, Madrid, Spain) and hydrogen peroxide (TraceSELECT™, Fluka™, Madrid, Spain) was added. The samples were digested in a Milestone Ultrawave microwave, and once digested were diluted to 25 mL with MilliQ water. The detection and quantification limits of Cu in the different matrices throughout the investigation are shown in the following Table 3.

### 2.9. Statistical Analysis

Data were processed with IBM SPSS 25.0 Statistics (IBM Corp., Armonk, NY, USA) and expressed as mean ± standard deviation. The normality of the variables was analyzed using the Shapiro-Wilk test. Student’s *t*-test was used to perform the comparisons in stretch stature, a one-way ANOVA to determine the differences along the assessments in female hormones and a two-way ANOVA (sex effect and measured effect) for the rest of the variables. For the measured effect variable, the Bonferroni post-hoc test was applied. Effect size was calculated using partial eta squared (η2), where 0.01–0.06 was a small effect size, 0.06–0.14 was a moderate effect size and >0.14 was a large effect size [35]. Differences of *p* < 0.05 and *p* < 0.01 were considered statistically significant and highly significant, respectively. The figures have been created using GraphPad 8 Software Inc (Boston, MA, USA).

## 3. Results

Table 4 shows the anthropometric characteristics, body composition and physical activity in the participants. There were significant differences in all anthropometric and body composition parameters between sexes (*p* < 0.001). Regarding differences between assessments, there were differences in the sum of skinfolds, fat and muscle (*p* < 0.05). Specifically, there were differences between assessments 1 and 2.

Table 5 shows the results obtained in the vertical jump test and the maximum incremental test to exhaustion. There were differences between sexes in all the parameters analyzed (*p* < 0.001). Moreover, there were differences between assessment 1 and 2 in maximum oxygen consumption (*p* < 0.05).

Table 6 shows the mean intakes for the three days of the macronutrients and Cu during the study. There were differences between sexes in the intakes of total energy and protein (*p* < 0.05), being higher in the men soccer players.

Table 7 compiles the erythrocyte and platelet values during the investigation. There were differences between sexes and between evaluations in the number of erythrocytes (*p* < 0.001). Differences between evaluations were found between evaluations 1 vs. 2 and 1 vs. 3 (*p* < 0.05).

Table 8 shows the values of female hormones in women soccer players throughout the study. There were no significant differences.

**Figure 1:**
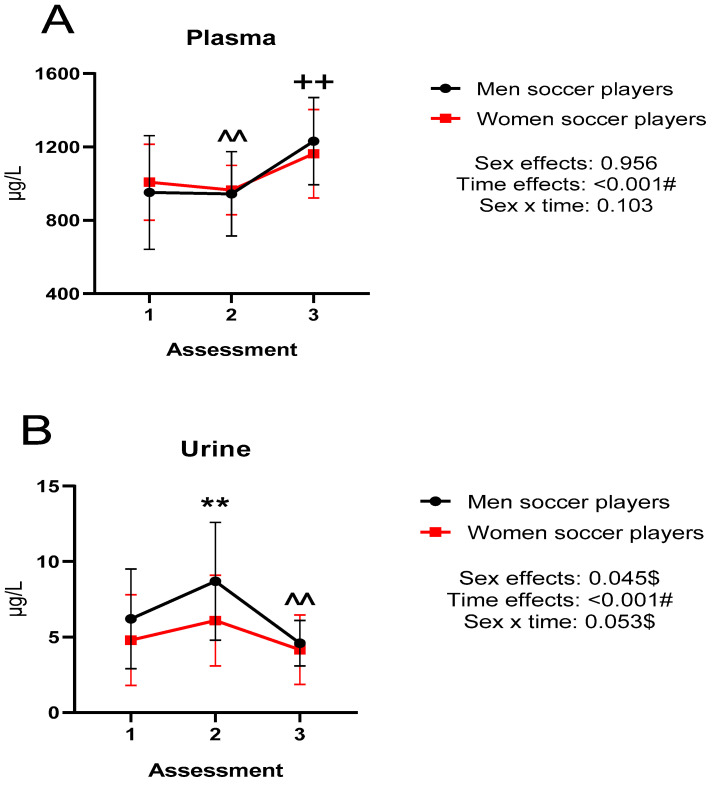
(**A**) plasma Cu concentrations; (**B**) urinary Cu concentrations; # large effect size (>0.14); $ moderate effect size (0.06–0.14); ** *p* < 0.01 differences between 1st and 2nd assessment; ++ *p* < 0.01 differences between 1st and 3rd assessment; ^^ *p* < 0.01 differences between 2nd and 3rd assessment; Cu: copper.

Figure 1 shows the extracellular (plasma and urine) copper concentrations during the investigation in both groups. There were differences between titrations with a large effect size (*p* < 0.001). Specifically, in plasma, differences were observed between titrations 1 vs. 3 and 2 vs. 3 (*p* < 0.01). In urine, there were differences between titrations 1 vs. 2 and 2 vs. 3 (*p* < 0.01). There were also significant differences between sexes in urine (*p* < 0.05).

**Figure 2:**
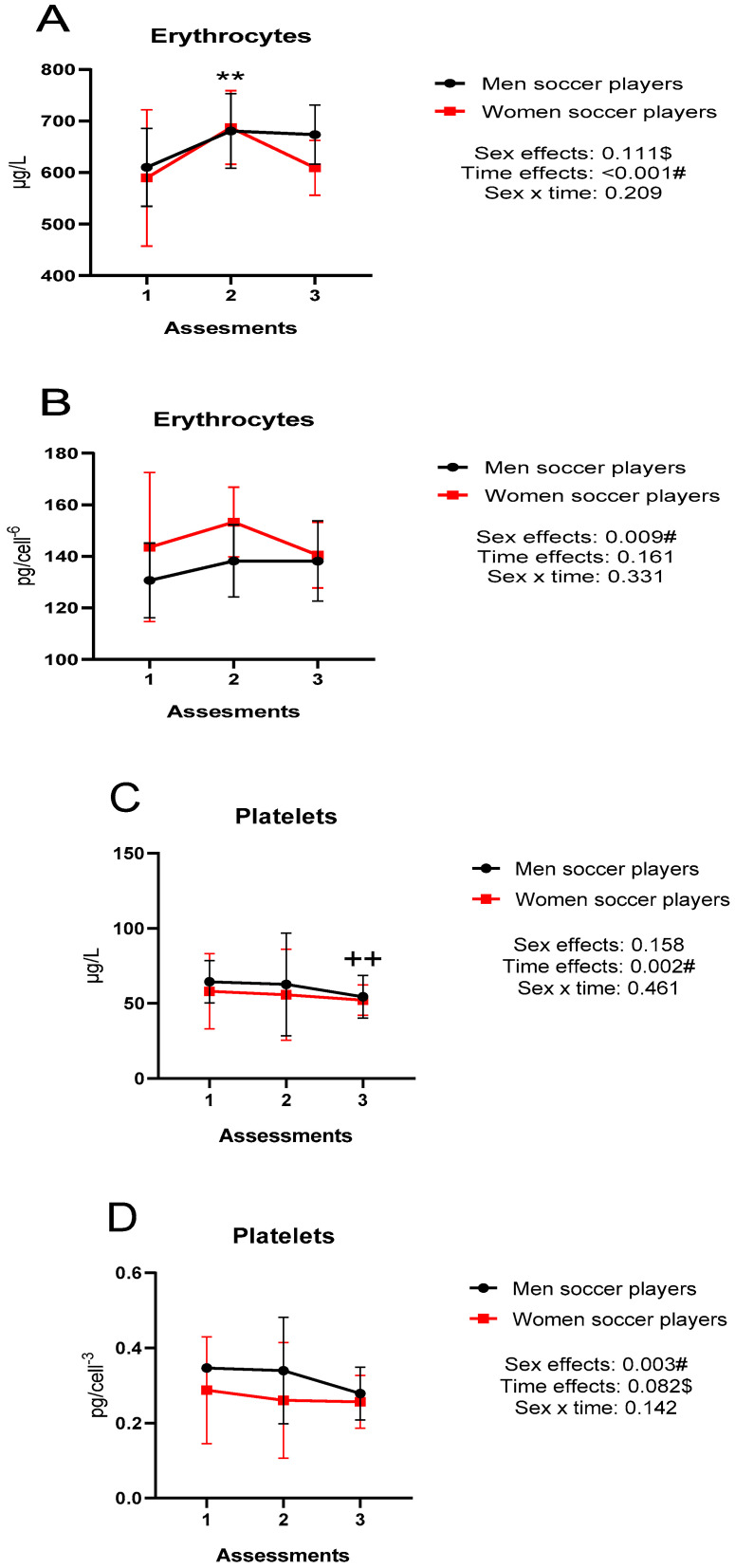
(**A**) absolute Cu concentrations in erythrocytes; (**B**) Cu concentrations in relation to the number of erythrocytes; (**C**) absolute Cu concentrations in platelets; (**D**) Cu concentrations in relation to the number of platelets; # large effect size (>0.14); $ moderate effect size (0.06–0.14); ** *p* < 0.01 differences between the 1st and 2nd assessment; ++ *p* < 0.01 differences between the 1st and 3rd assessment; Cu: copper.

Figure 2 represents the intracellular concentrations (erythrocytes and platelets), in absolute values and relative to the number of cells, throughout the investigation. There were sex differences in erythrocyte and platelet Cu concentrations expressed in relative values (*p* < 0.01). As for differences throughout the study, there were differences in absolute Cu concentrations in both platelets and erythrocytes (*p* < 0.01). In erythrocytes (A), there were differences between titration 1 and 2 (*p* < 0.01), while in platelets (C) there were differences between titration 1 and 3 (*p* < 0.01).

## 4. Discussion

The objectives of the research were (i) to analyze changes in intracellular and extracellular Cu concentrations during a season in soccer players and (ii) to analyze sex differences. Among the novelties of the present study are the longitudinal comparison of Cu concentrations in the different biological matrices simultaneously and the comparisons between sexes in athletes. Studies that have investigated the influence of exercise on Cu concentrations in athletes have analyzed one [36,37,38], two [12,39] or three compartments [40]. Recently, Toro-Román et al. [20] studied Cu concentrations in up to five compartments in soccer players to obtain a complete assessment, observing important discrepancies between biological matrices. The Cu concentrations detected in each compartment are within the ranges reported in other investigations where they determined such concentrations with similar techniques, both in a general population [41,42,43] and differentiating between men [12,36] and women [44,45].

In addition to reproductive function, female sex hormones affect numerous cardiovascular, respiratory, thermoregulatory, and metabolic parameters, with implications for exercise physiology [46]. As shown in Table 8, the assessments were performed at approximately the same phase of the menstrual cycle in the women soccer players. This is key since the menstrual cycle could influence Cu concentrations [27,47].

Cu plays an important role for health as it is involved in various biological processes [48]. Therefore, Cu has been the subject of intense research for several decades. Despite this, there is no consensus on the ideal biomarker to analyze its status [49]. Various indices such as cuproenzymes, serum Cu or plasma Cu have been routinely analyzed in human studies [18]. Most current approaches use cuproenzymes. However, the responsiveness and concentration of ceruloplasmin may be affected by inflammation. Ceruloplasmin is a protein regulated by inflammatory hormones, and thus individuals with chronic inflammatory conditions may have elevated levels [7]. Intervention studies have shown little or no effect of short-term Cu deficiency on ceruloplasmin levels or activity [50]. However, its activity has been reported to decrease in response to severe Cu deficiency [51]. The review by Harvey et al. [18] concluded that serum Cu may be the most useful biomarker of Cu status and appears to be effective in both replenished and depleted individuals. However, a change in a single index may be inadequate to assess nutritional status [19]. In view of the above, in the present study we chose to perform a multicompartmental analysis in order to have data from different biological matrices and to discover the Cu status. In addition, intracellular Cu analysis is not common in the scientific literature [38,47], so this investigation could provide reference data for future studies. It is known that the half-life of erythrocytes is approximately 120 days [52], whereas platelets have a half-life of approximately 10 days [53]. Therefore, erythrocytes could provide outdated information, contrary to platelets. This is one of the few studies analyzing platelet Cu concentrations in athletes.

It is important to assess TME intake as TME status could estimate physical performance [54]. Cu homeostasis is fundamental in physical activity and sport [4]. Food intake is the main source for obtaining Cu [55]. In the present study, both groups ingested higher amounts of Cu than the dietary reference intakes (DRI = 1100 μg/day) [56]. Cu intakes in the women soccer players were lower than those reported by Nuviala et al. [44] in women athletes of different sports modalities. However, they were higher when compared with another group of women soccer players [57]. Regarding men soccer players, Toro-Román et al. [20] reported a higher intake of Cu. Similarly, Koury et al. [58] reported higher Cu intakes in triathletes and runners of different distances.

Firstly, taking into consideration the changes along the evaluations (longitudinal), it was observed in both groups that plasma Cu concentrations decreased in the second evaluation and increased at the end of the study above the initial values. There is little research studying the chronic effect of physical training. In long-distance and middle-distance athletes, after six months of training, there was a decrease in serum Cu concentrations [12]. In basketball players, no significant changes in plasma Cu concentrations were observed after three weeks of high-intensity training [59]. Similarly, in swimmers, Lukaski et al. [23] observed no changes in plasma Cu concentrations when analyzing both men and women at the beginning and at the end of the season.

The increases in Cu observed at the end of the investigation could be due to different reasons. On the one hand, an increase in Cu intake [19]. On the other hand, an increase in plasma concentrations of ceruloplasmin due to its antioxidant properties [60,61], since during a period of training there is an increase in free radicals and oxidative damage [62]. Increased ceruloplasmin production could be an adaptive response of the organism to an increased need for Cu. Cu is crucial for maintaining cytochrome c oxidase activity and cellular receptor sites for ceruloplasmin have been identified [20,61]. Cu is also known to be important for SOD formation [58]. An increase in SOD activity could represent an adaptive response to intracellular oxidative stress induced by training [23].

In urine, contrary to plasma concentrations, there was an increase in the second titration. In the third titration the concentrations decreased and became lower than in the first titration. In contrast to the present study, Maynar et al. [12] observed lower Cu excretions in long-distance athletes after six months of training. When the acute effect of physical exercise was analyzed, decreases in Cu excretion were observed in athletes [39]. Granell [63] observed increases in Cu excretion after two types of training (aerobic endurance and muscular strength).

Cu is under homeostatic controls due to the pathological consequences of dyshomeostasis [64]. The increase in Cu excretion could indicate an increase in Cu metabolism in order to balance the energy and anti-oxidant demands as a consequence of physical training [65]. However, the decrease in urinary excretion could be an adaptive measure to avoid a decrease in body Cu content in order to favor adaptive processes of training [39]. This would be in agreement with the results obtained in plasma.

Regarding the absolute concentrations of Cu in erythrocytes, increases were observed in both groups in the second evaluation. It should be noted that no studies have been found that evaluate changes in erythrocyte Cu concentrations over time. The studies related to this subject are based on analyzing baseline differences. In relation to the above, Maynar et al. [38] reported that subjects with moderate and high levels of physical training had lower erythrocyte Cu concentrations compared to sedentary subjects. Recently, Toro-Román et al. [20] showed lower erythrocyte Cu concentrations in soccer players. In women, Singh et al. [47] reported that women runners showed lower erythrocyte Cu concentrations compared to sedentary women.

The increase of Cu in erythrocytes could be due to increases in ROS generated during physical training. Increased ROS stimulate antioxidant mechanisms in both muscle and erythrocytes [58]. As a consequence, SOD in erythrocytes increases its activity [66]. Therefore, the increase of Cu in erythrocytes would be related to a possible increase of SOD activity to counteract the increase of free radicals [67].

Regarding the variations throughout the investigation of intraplatelet Cu concentrations, significant differences were observed in the third evaluation. It is not common to analyze TME in this type of compartment. In athletes, it has recently been observed that physically active subjects have lower Cu concentrations compared to sedentary subjects [20]. Most of the studies found comparing platelet Cu concentrations had as subjects people with pathologies [68,69]. Unfortunately, due to the scarce information on platelet Cu concentrations in athletes, we cannot justify the changes produced during the study.

Secondly, taking into account the differences between sexes in each of the biological parameters analyzed, no differences were observed between sexes in plasma Cu concentrations. These results are similar to those reported previously [23,70]. However, Milne and Johnson [71] observed significant differences between sexes divided by different age ranges, being higher in most ranges in women. Similarly, García et al. [72] observed higher concentrations in women in all seasons compared to men. Other authors noted that women were exposed to higher levels of Cu than men [73]. Previous authors reported that hormonal differences between sexes could influence Cu concentrations. Endogenous hormones can affect Cu metabolism at the cellular and body level [74]. Animal studies suggest that higher estrogen levels may enhance the production of ceruloplasmin, thereby increasing Cu absorption and concentrations [73]. However, some studies that reported higher plasma and serum Cu concentrations in women also reported their taking oral contraceptives [15]. In the present study no participant claimed to ingest any contraceptive method. Therefore, we believe that this fact influenced the absence of significant differences between sexes. Also, unlike the previous studies cited, the population of the present study were athletes, which could be a factor to be considered.

In urine, significant sex differences were reported in the present study. Specifically, men excreted higher amounts of Cu compared to women. In relation to the above, similar results have previously been observed in a general population, without being significant [75]. However, a study published in 1994 reported in a general Spanish population from different demographic areas that women excreted greater amounts of Cu regardless of the area of residence [76], as did another study by Folch et al. [77]. The difference between sexes in urinary Cu could be related to the biological mobilization of this mineral induced by physical training [78]. It is known that the intensity and load of matches is higher in men soccer players compared to women soccer players [79]. Differences in the physical demands of training and matches could influence Cu excretion.

Regarding intracellular concentrations, significant differences between sexes have been observed in erythrocyte Cu concentrations, being higher in women, expressed in relative values. However, these differences disappear when the values are expressed in absolute values. Hinks et al. [80] reported no sex differences in erythrocyte Cu concentrations. However, in a Spanish and Japanese population aged 7 to 11 years, they observed that girls showed higher erythrocyte Cu [81]. Women might have a higher erythrocyte Cu uptake than men [71], which could influence the Cu count. It should be noted that, after observing the results, it is important to express Cu concentrations in absolute and relative values since, due to the lower number of erythrocytes characteristic in women, the data may be misinterpreted.

As for platelet Cu concentrations, decreases in concentrations were observed throughout when the results were expressed in absolute values. However, when the values were expressed relative to the number of platelets, there were significant differences between sexes (*p* < 0.05). We have not found studies that justify this fact.

The present investigation is not free of possible limitations: (i) The absence of complementary data (e.g., enzymes); (ii) the small number of the sample; (iii) technical measurement error was not analyzed and (iv) the absence of studies on the differences between sexes in Cu concentrations in different matrices complicates the discussion and justification of the results.

## 5. Conclusions

During a sports season, changes in extracellular and intracellular Cu concentrations occur in soccer players. Specifically, plasma concentrations increase at the end of the season while urinary concentrations increase in mid-season and decrease at the end of the season. However, platelet concentrations gradually decrease during the season while erythrocyte concentrations rise in mid-season and are maintained until the final period.

Urinary, erythrocyte concentrations in relation to cell number, and platelet concentrations in relation to cell number, were different between sexes.

The assessment of TME status during a season is important to know the possible need to incorporate nutritional supplementation because TME deficiency could negatively influence performance if maintained for long periods. Due to the discrepancies, it is important to use various markers to assess Cu status.

## Figures and Tables

**Table 1:** Characteristics of soccer players.

		Men Soccer Players	Women Soccer Players
N		22	24
Age (years)		20.62 ± 2.66	23.21 ± 4.11
Experience (years)		14.73 ± 3.13	14.51 ± 4.94
Position on the field (%)	Goalkeeper	7.70	11.10
	Defender	30.80	33.30
	Midfielder	38.50	29.60
	Forward	23.10	25.90
Total Training (weeks)		36	39
Total Training (n°)		128.27 ± 18.59	133.54 ± 25.86
Total Training (min)		11,814.23 ± 1673.40	10,578.46 ± 3227.80
Absence from training (days)		12.07 ± 9.34	14.14 ± 10.79

**Table 2:** Characteristics of the menstrual cycle of the women soccer players.

	Women Soccer Players
Age of onset (years)	13.5 ± 1.15
Regular menses (%)	100.00
Duration of bleeding (days)	4.77 ± 1.47
Menstrual cycle (days)	27.93 ± 2.78
Use of contraceptive methods (%)	0

**Table 3:** Limits of detection and limits of quantification for Cu.

Matrix	Limits of Detection (µg/L)	Limits of Quantification (µg/L)
Plasma	0.005	0.05
Urine	0.012	0.12
Erythrocytes	0.002	0.02
Platelets	0.010	0.10

**Table 4:** Anthropometric characteristics, body composition and physical activity of the participants.

		Men Soccer Players	Women Soccer Players	Sex Effect	Time Effect	Sex × Time
Stretch stature (m)	1st assessment	1.76 ± 0.061	1.65 ± 0.06 ^++^	-	-	-
	-	-	-			
	-	-	-			
Body mass (kg)	1st assessment	71.50 ± 5.93	59.58 ± 7.17	<0.001	0.748	0.931
	2nd assessment	71.95 ± 5.87	60.44 ± 6.77			
	3rd assessment	72.80 ± 5.68	66.39 ± 8.99			
Σ6 Skinfold (mm)	1st assessment	60.34 ± 12.35	94.62 ± 18.54	<0.001	0.009	0.016
	2nd assessment	60.12 ± 12.61	76.72 ± 15.13 *			
	3rd assessment	56.85 ± 12.12	83.81 ± 18.75			
Fat (%)	1st assessment	9.46 ± 1.30	18.16 ± 2.74	<0.001	0.005	0.007
	2nd assessment	9.45 ± 1.31	15.56 ± 2.16 *			
	3rd assessment	9.14 ± 1.23	16.54 ± 2.68			
Muscle (%)	1st assessment	50.80 ± 1.67	45.62 ± 3.03	<0.001	0.022	0.013
	2nd assessment	50.62 ± 1.34	48.36 ± 2.76 *			
	3rd assessment	51.04 ± 1.28	47.54 ± 3.54			
Total PA (MET min/week)	1st assessment	5646 ± 2917	6271 ± 3801	0.555	0.123	0.144
	2nd assessment	5596 ± 2063	5851 ± 2751			
	3rd assessment	5644 ± 3026	6051 ± 2961			

* *p* < 0.05 differences between 1st and 2nd assessments; ++ *p* < 0.01 differences between groups (Student’s *t* test). BMI: body mass index; Σ; sum; PA: physical activity; MET: Metabolic Equivalent of Task.

**Table 5:** Results obtained in the vertical jump and in the maximum incremental test of the participants.

		Men Soccer Players	Women Soccer Players	Sex Effect	Time Effect	Sex × Time
SJ (cm)	1st assessment	50.52 ± 6.48	35.65 ± 5.82	<0.001	0.515	0.602
	2nd assessment	49.73 ± 4.21	37.08 ± 5.14			
	3rd assessment	50.90 ± 6.2	38.00 ± 5.49			
CMJ (cm)	1st assessment	56.94 ± 6.39	40.21 ± 7.46	<0.001	0.571	0.717
	2nd assessment	55.34 ± 4.72	39.70 ± 4.18			
	3rd assessment	56.05 ± 6.39	41.45 ± 5.80			
Maximum speed (km/h)	1st assessment	19.17 ± 1.72	15.73 ± 1.16	<0.001	0.289	0.315
	2nd assessment	19.22 ± 1.44	15.20 ± 1.10			
	3rd assessment	19.15 ± 1.98	14.91 ± 1.37			
VCO_2max_ (L/min)	1st assessment	4.05 ± 0.36	2.68 ± 0.44	<0.001	0.377	0.710
	2nd assessment	3.85 ± 0.80	2.64 ± 0.31			
	3rd assessment	3.87 ± 0.41	2.61 ± 0.30			
VO_2max_ (mL/min/kg)	1st assessment	52.21 ± 2.91	39.72 ± 6.22	<0.001	0.032	0.268
	2nd assessment	54.79 ± 3.70 *	42.32 ± 4.19 *			
	3rd assessment	53.30 ± 5.11	41.06 ± 4.51			
HR_max_ (lpm)	1st assessment	187.78 ± 6.52	183.33 ± 7.34	<0.001	0.177	0.204
	2nd assessment	188.90 ± 5.82	179.75 ± 8.11			
	3rd assessment	186.90 ± 7.42	176.90 ± 8.00			

* *p* < 0.05 differences between 1st and 2nd assessments; SJ: squat jump; CMJ: counter movement jump; HR: heart rate; VO_2max_: maximum oxygen consumption; VCO_2max_: maximum carbon dioxide volume.

**Table 6:** Mean intake of the three days of evaluation of macronutrients and Cu throughout the study.

		Men Soccer Players	Women Soccer Players	Sex Effect	Time Effect	Sex × Time
Energy (Kcal)	1st assessment	1796.0 ± 420.0	1578.1 ± 316.2	0.038	0.497	0.317
	2nd assessment	1932.2 ± 312.5	1681.5 ± 427.3			
	3rd assessment	1882.7 ± 358.6	1697.3 ± 386.1			
Proteins (g/day)	1st assessment	106.1 ± 25.5	90.4 ± 21.6	0.047	0.469	0.218
	2nd assessment	115.5 ± 23.4	96.2 ± 18.3			
	3rd assessment	108.9 ± 24.8	92.6 ± 20.4			
Lipids (g/day)	1st assessment	54.8 ± 19.1	48.3 ± 12.3	0.116	0.241	0.471
	2nd assessment	64.1 ± 15.4	55.6 ± 15.3			
	3rd assessment	58.6 ± 17.4	60.3 ± 20.6			
Carbohydrates (g/day)	1st assessment	231.0 ± 69.1	206.1 ± 81.3	0.471	0.856	0.683
	2nd assessment	235.8 ± 60.3	241.5 ± 56.1			
	3rd assessment	242.0 ± 57.0	235.8 ± 61.7			
Cu (µg/day)	1st assessment	1267.3 ± 494.6	1341.6 ± 502.4	0.489	0.688	0.576
	2nd assessment	1331.3 ± 399.0	1401.5 ± 431.8			
	3rd assessment	1315.6 ± 502.6	1415.8 ± 491.0			

Cu: copper.

**Table 7:** Erythrocyte and platelet values according to sex throughout the study.

		Men Soccer Players	Women Soccer Players	Sex Effect	Time Effect	Sex × Time
Erythrocytes (millions)	1st assessment	4.92 ± 0.36	4.37 ± 0.22	<0.001	0.031	0.063
	2nd assessment	4.83 ± 0.32 **	4.19 ± 0.27 **			
	3rd assessment	4.99 ± 0.29 ^++^	4.35 ± 0.27 ^++^			
Platelets (thousands)	1st assessment	204.50 ± 57.65	196.00 ± 38.01	0.274	0.542	0.222
	2nd assessment	196.60 ± 39.79	219.08 ± 34.19			
	3rd assessment	195.13 ± 37.82	204.39 ± 31.52			

** *p* < 0.01 differences between 1st and 2nd assessment; ++ *p* < 0.01 differences between 1st and 3rd assessment.

**Table 8:** Female hormones throughout the sports season.

		Women Soccer Players	*p*
Progesterone (ng/mL)	1st assessment	2.65 ± 3.88	0.998
	2nd assessment	2.38 ± 3.21	
	3rd assessment	2.31 ± 2.89	
Estradiol-17β (pg/mL)	1st assessment	74.04 ± 45.30	0.894
	2nd assessment	71.32 ± 39.25	
	3rd assessment	68.30 ± 40.93	

## Data Availability

Not applicable.
